# Ultrasonic Multiple-Access Ranging System Using Spread Spectrum and MEMS Technology for Indoor Localization

**DOI:** 10.3390/s140203172

**Published:** 2014-02-18

**Authors:** Laurent Segers, Jelmer Tiete, An Braeken, Abdellah Touhafi

**Affiliations:** 1 Department of Industrial Sciences and Technology (INDI), Vrije Universiteit Brussel, Pleinlaan 2, Elsene 1050, Belgium; E-Mails: an.braeken@vub.ac.be (A.B.); abdellah.touhafi@vub.ac.be (A.T.); 2 Department of Electronics and Informatics (ETRO), Vrije Universiteit Brussel, Pleinlaan 2, Elsene 1050, Belgium; E-Mail: jelmer.tiete@etro.vub.ac.be

**Keywords:** ultrasound wideband indoor localization, ultrasound multiple-access ranging, ultrasound FHSS using finite impulse response filtering, ultrasound DSSS

## Abstract

Indoor localization of persons and objects poses a great engineering challenge. Previously developed localization systems demonstrate the use of wideband techniques in ultrasound ranging systems. Direct sequence and frequency hopping spread spectrum ultrasound signals have been proven to achieve a high level of accuracy. A novel ranging method using the frequency hopping spread spectrum with finite impulse response filtering will be investigated and compared against the direct sequence spread spectrum. In the first setup, distances are estimated in a single-access environment, while in the second setup, two senders and one receiver are used. During the experiments, the micro-electromechanical systems are used as ultrasonic sensors, while the senders were implemented using field programmable gate arrays. Results show that in a single-access environment, the direct sequence spread spectrum method offers slightly better accuracy and precision performance compared to the frequency hopping spread spectrum. When two senders are used, measurements point out that the frequency hopping spread spectrum is more robust to near-far effects than the direct sequence spread spectrum.

## 1. Introduction

Indoor localization becomes a ever more real phenomenon in everyday life. One might think of indoor cleaning robots, patient tracking in hospitals, *etc.* The global positioning system (GPS) already assists people in outdoor situations, however, for indoor situations, GPS does not work or performs badly. Techniques like received signal strength indicator (RSSI), time of arrival (ToA), angle of arrival (AoA) and time difference of arrival (TDoA) try to overcome this limitation. RSSI [1,2] uses the signal strength of radio communication to estimate the distance between devices. The AoA [3,4] technique uses an array of receivers to evaluate the incoming reception angle. Calculating the location of the source is done by combining the angles of different receivers. In ToA [5], nodes try to estimate distances by evaluating the round trip time of a message and its reply. TDoA uses a radio synchronization packet followed by an ultrasonic pulse. The distance between nodes is calculated by the travel time of the ultrasound signal. This last method also offers the highest level of accuracy for indoor localization [6].

Within TDoA, two ultrasound ranging categories have been proposed. The first approach consists of sending a simple narrowband pulse, which is also known as the impulsive technique. A second approach consists of spreading the signal using orthogonal codes. Direct sequence and frequency hopping spread spectrum signals have previously been introduced in ultrasound ranging techniques [7,8]. The frequency hopping spread spectrum (FHSS) and direct sequence spread spectrum (DSSS) offer the advantage of being more noise resistant compared to non-spread spectrum techniques. Both methods have their own (dis)advantages [9]. DSSS uses a signal carrier on which an orthogonal spreading code is modulated together with the data. This results in a signal being spread around the carrier. By contrast, the FHSS method uses a carrier that switches between a given set of frequencies. The followed frequency pattern depends an a given orthogonal code. The former method is likely to be more resistant to white noise, while the latter method is more resistant to in-band noise.

In this work, a novel ranging approach using the frequency hopping spread Spectrum with a finite impulse response (FIR) filter will be developed and compared against the direct sequence spread spectrum. The senders of both DSSS and FHSS will be implemented using field programmable gate arrays (FPGAs). The outline of this paper will be as follows: Section 2 will give an overview of existing systems within the context of the time difference of arrival. In Section 3, our system setup will be detailed. Section 4 will review our measurement results, followed by concluding notes and future work in Section 5.

## 2. Related Work

There are a number of TDoA-based indoor localization systems described in the literature. In the Cricket [10] and the Bat [11] systems, nodes use the time of flight of a narrowband ultrasonic pulse to calculate their position. The topology of these systems is composed of fixed nodes or beacons and mobile nodes, where the mobile nodes try to calculate their position based on the fixed nodes. These systems offer a high level of accuracy. However, the disadvantage is the considerable amount of fixed position nodes needed, which increases the setup cost. To overcome this, Fukuju Yasuhiro *et al.* [12] developed the Dolphin system, which uses an incremental approach. In this approach, a smaller amount of fixed position nodes are deployed, from which the other nodes can calculate their position. The incremental approach results in a less accurate localization than Cricket and BAT.

Other systems use a wideband ultrasound approach to calculate distances between nodes [13,14]. The wideband approach mainly consists of modulating a unique code or identifier for each node onto a carrier. This technique requires transducers that are able to operate in a certain frequency bandwidth. Mike Hazas and Andy Ward use a polyvinylidene fluoride (PVDF) film placed in a cylindrical fashion to send and receive wideband ultrasound modulated signals [13]. A Gold code is modulated on a carrier using binary phase shift keying (BPSK). The PVDF film setup is designed to operate at frequencies between 20 kHz and 100 kHz; however, this transducer needs a high operating voltage. Hernandez *et al.* proposed another approach using narrowband piezo crystal transducers [15]. The approach consists of compensating for the small bandwidth of the transducer by adding an inductor-resistor circuit. An ultrasonic MEMS transducer is then used to receive the generated waveforms. Their results show a bandwidth increase from 2 kHz to 15kHz. Omar *et al.* modulated a 63-bit Gold code with a baud rate of 2 kHz onto a 40.2-kHz carrier using piezo crystal transducers with an accuracy of a few centimeters [14]. In [7], FHSS has been simulated against DSSS, whereas in [8], FHSS has been used in combination with the angle of arrival. The latter system uses an array of MEMS microphones and calculates the reception angle with the multiple signal classification algorithm (MUSIC).

The accuracy level of the measurements of narrowband [14] systems is generally about a few centimeters. With the incremental approach, the accuracy drops to about 15 cm [13]. The advantage of these systems is the simple ultrasonic interface and low computational needs. However, the narrowband ultrasonic pulses are vulnerable to noise, and only one sender is allowed to send a pulse in a given time slot. With the wideband approaches, these problems can be alleviated. Existing systems using this technique are able to achieve sub-centimeter accuracies, but with a higher processing power cost.

## 3. Architectural Design

The architecture is composed of a set of senders and receivers. The senders emit orthogonal ultrasound coded signals, which are received at the receiver side. The receiver will use a real-time time-correlator to retrieve the flight time of the received and sampled signals. In our first setup, we have used a physical wire to create an accurate time-synchronization between sender and receiver. This allows us to retrieve the upper bound of the achievable accuracy. The implementation aspects of the different parts of our setup are detailed in the following sections.

## 3.1. Hardware

**Figure 1.**
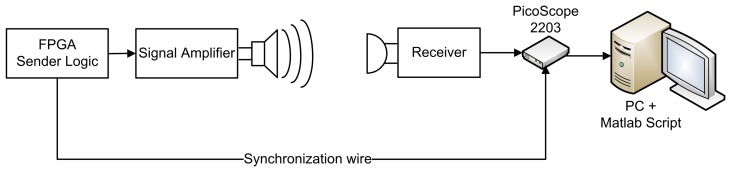
Setup consisting of one sender and one receiver. FPGA, field programmable gate array.

In order to be able to send and receive ultrasound signals, both sender and receiver are equipped with ultrasound transducers. We choose the ultrasound SPM0404UD5 MEMS sensor [16] as the receiver transducers. This sensor is able to measure sound waves with frequencies ranging from 10 kHz to 65 kHz. This sensor offers omnidirectional sensing capabilities and has a small footprint on a printed circuit board (PCB). Although it has a broad frequency range, only a part will be used in our experiments. Sound signals with frequencies below the 20 kHz threshold may interfere with human hearing and are therefore not suitable. Due to the specifications of the transducer, signals with frequency components beyond the 65 kHz boundary are also not desired, since they may cause signal aliasing. In order to prevent aliasing and low frequency noise, the input signal is filtered through a bandpass filter with cut-off frequencies situated at 20 kHz and 60 kHz. The input signal from the sensor is also amplified in order to compensate for the decrease in the received signal strength due to the distance between sender and receiver. A PC oscilloscope (PicoScope 2203) digitizes the signal at a sample rate of 250 kSps. The signal is then processed by a MATLAB script on a computer (Figure 1).

At the sender side, we use the Senscomp Instrumental Grade 600 Electrostatic transducer [17]. This transducer has an operating frequency ranging up to 100 kHz. However, the best frequency responses can be achieved in the 50 kHz to 65 kHz range. In our setup, we noticed operating frequencies ranging from 25 kHz up to 60 kHz to be well received by the receiver. An element of attention for this transducer is the small beam angle of 15 degrees. A small change in alignment causes an almost complete signal loss. Although the transducers’ optimal bias and amplitude voltage for a 50 kHz signal are respectively at 200 V and 400 V peak-to-peak . Due to the wideband signals in our system, these settings cause a strong signal degradation with increasing frequency, which is probably due to impedance matching problems with wideband signals. To reduce this degradation, both the bias and amplitude voltage have been reduced to 120 V and 160 Vpeak-to-peak. A transistor circuit amplifies the signal from the sender logic to the 160 Vpeak-to-peak for the electrostatic transducer.

### 3.2. Signal Generation

Frequency hopping (FHSS) and the direct sequence spread spectrum (DSSS) have previously been introduced. In our implementation, the sender logic of both FHSS and DSSS is done on a field programmable gate array (FPGA). FPGAs are digital reconfigurable chips on which digital circuit designs can be implemented. FPGAs also allow designers to implement circuits with a time accuracy of one clock tick (*i.e.*, 20-ns time accuracy with a 50-MHz clock). The FHSS and DSSS sender logic have been designed in the very high speed integrated circuit hardware description language (VHDL) with the Xilinx integrated software environment (ISE). The chosen FPGA platform is the Suzaku-S [18] board with the Xilinx Spartan 3 XC3S400-FT256 FPGA. This board is clocked at 3.6864 MHz and has a large amount of FPGA input-output (IO)-pins. FHSS and DSSS senders are implemented separately, which means that at any given time, only one of the two methods is implemented on the FPGA.

DSSS and FHSS use spreading techniques in order to make the transmitted data more robust to noise and signal collisions. The DSSS technique uses binary phase shift keying to enlarge the transmitted signal spectrum (*i.e.*, to spread), while FHSS uses a carrier that switches between a given set of frequencies.

#### 3.2.1. DSSS

DSSS is characterized by data that is modulated onto a carrier. The result is spread with an orthogonal code using binary phase shift keying (BPSK). In our setup, we use Gold code as the orthogonal code. The length of the Gold code is set to 63 symbols. The transmission time of one symbol is about 500 μs, while the total sending time is 31.5 ms. The signal is repeated every 63 ms (*i.e.*, the time for sending one frame).

Time correlation is done using the orthogonal code. Therefore, the data sent in our setup is always a logic ‘1′, meaning there is no data sent. This latter also simplifies the sending logic.

**Figure 2.**
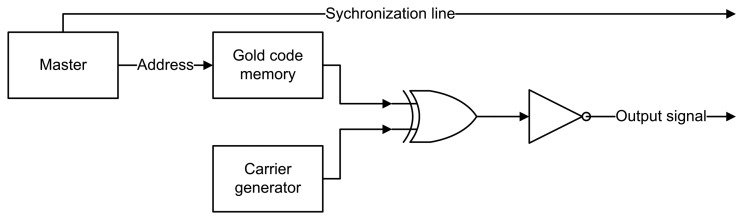
DSSS sender logic.

An overview of the DSSS sender logic on FPGA is given in Figure 2. The carrier is a square wave with a frequency of 40.960 kHz. The Gold code is stored as a collection of ‘1′ and ‘0′ instead of ‘1′ and ‘−1′ in a Gold code memory. Accessing a certain symbol in this memory is done by using an index (address).

The modulation process of the Gold code onto the carrier is given in truth Table 1. The result of the truth table can be written as:

(1) $$O{=}^{\neg }(C\underline{\vee }G)$$

**Figure 3.**
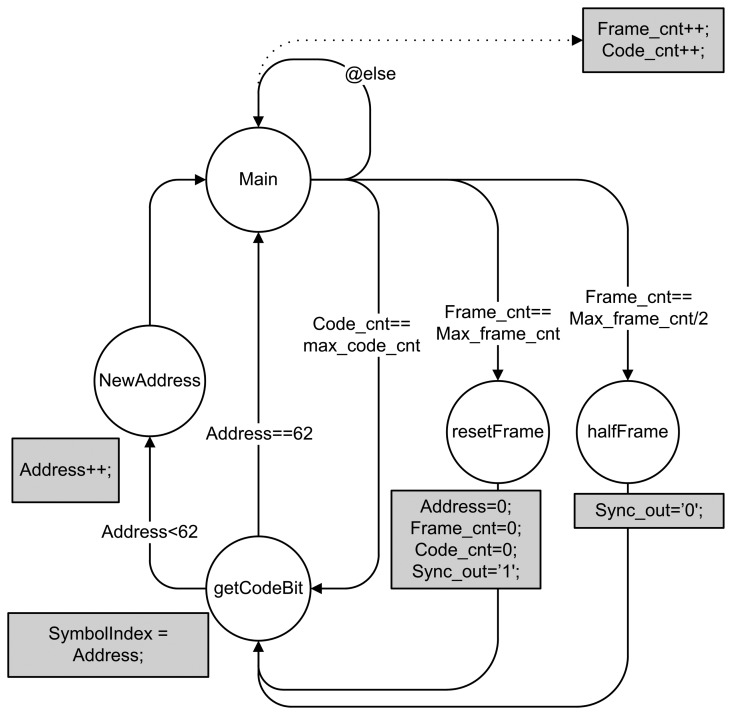
Finite state machine of the master module.

Next to the carrier generator and Gold code memory, one can also find the synchronization mechanism or master module. The reason for the synchronization mechanism is two-fold. On the one hand, the sender and receiver are perfectly synchronized through the synchronization wire. On the other hand, this module also ensures correct timing for the Gold code memory and symbol address generation. A finite state machine (FSM) of the master module is given in Figure 3. The Max_frame_cnt and max_code_cnt respectively correspond to the total duration of one frame and to the duration of one symbol. The output signals are Sync_out (synchronization wire) and SymbolIndex (address in Figure 2).

#### 3.2.2. FHSS

In FHSS, data are sent along with a carrier that switches between different frequencies at a given time interval. The switch (or hop) between different frequencies is done by using Gold code. A two-dimensional variant of the code is being used in order to be able to hop between four frequencies. Like in DSSS, the length of the Gold code is set to 63 symbols. The time for the sending of one symbol is about 500 μs, while the total sending time is 31.5 ms. The signal is repeated every 63 ms (*i.e.*, the duration of one frame).

**Figure 4.**
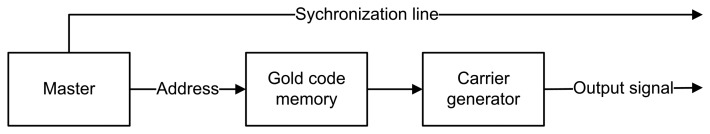
Frequency hopping digital logic.

Figure 4 gives an overview of the modules used in FHSS. As in DSSS, no data is being sent, and thus, the data remains constant (*i.e.*, logic ‘1′). The Gold code memory modules and carrier generator modules are slightly different from those in DSSS. The output of the Gold code memory determines on which frequency the carrier generator will be set. The four carrier frequencies were chosen at 36.864 kHz, 40.960 kHz, 46.080 kHz and 51.200 kHz. The chosen frequencies are whole dividers of the 3.6864 MHz oscillator and are sufficiently spaced from each other in the frequency domain.

#### 3.2.3. DSSS and FHSS Implementation Cost and Output Result

The sender logic of both FHSS and DSSS is implemented onto an FPGA. The chosen FPGA is the Xilinx Spartan XC3S400-FT256, which contains 7,168 lookup tables (LUTs), 3,584 slices (group of LUTs) and 173 input-output (IO) buffers. The amount of the used IO buffers remains constant regardless of the implementation (Table 2). The IO buffers are used to connect the internal logic with the physical IO pins of the FPGA chip. In our case, these are used for the input clock, the synchronization line and the output signal. The output signal corresponds to the ultrasonic coded signal to be sent.

**Figure 5.**
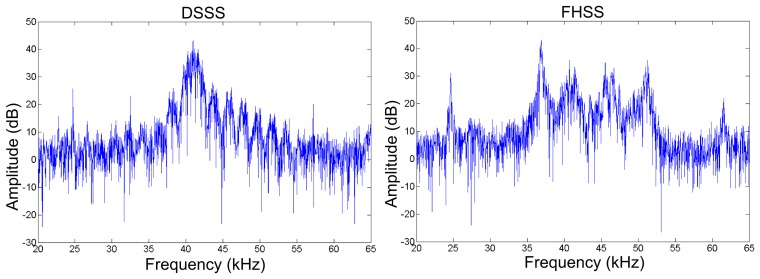
Frequency spectra of DSSS and FHSS measured by the receiver at 100 cm from one sender, peaking once at 40.960 kHz (DSSS), and four times at 36.864 kHz, 40.960 kHz, 46.080 kHz and 51.200 kHz (FHSS).

Both implementations use less than 2% of the available logic area. However, the FHSS method uses more logic, which is due to the increased complexity in the way in which the carrier is generated. The FPGA could be fully utilized with a 62-channel FHSS sender or a DSSS sender with 71 channels. The required IO buffers for FHSS and DSSS would respectively increase to 125 and 145, where the input clock would be taken as a common line for all channels. The resulting output signal of the DSSS and FHSS methods received at 100 cm from one sender is given in Figure 5.

### 3.3. Ranging Calculation

Ranging calculations are done by using the time correlation of the input signal against a reference signal. The input signal is the ultrasound coded signal obtained from the ultrasound MEMS transducer. The signal itself can be either DSSS or FHSS coded. The time correlation process differs for both methods.

#### 3.3.1. DSSS Ranging

**Figure 6.**
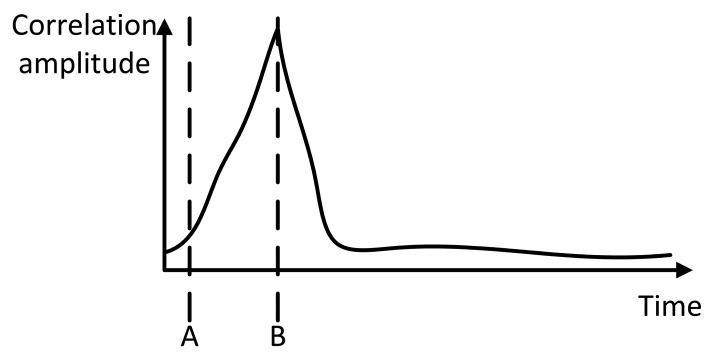
Expected correlation shape.

**Figure 7.**
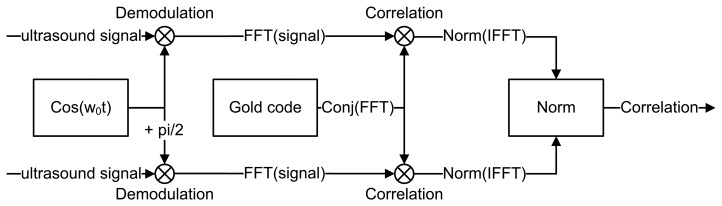
Direct sequence spread spectrum signal correlator.

The direct sequence spread spectrum method has been studied in [13,14]. The DSSS ranging method uses a dual channel correlator. Both channels are first demodulated by multiplying the complete input sample sequence with the original carrier. The length of the complete input sequence corresponds to the length of one frame (*i.e.*, 63 ms). One of the two input branches has a phase shift of $\frac{\pi }{2}$ radians, while the other remains in phase. The combination of both resulting correlations ensures a reliable reading, regardless of the phase shift of the input signal. Signal correlation is applied using the following formula:

(2) Corr=IFFT(FFT(S)⋅Conj(FFT(R)))

where *Corr*, *S* and *R* respectively represent the correlation result, the input signal and the reference signal. *IFFT* and *Conj* respectively denote the inverse fast Fourier transform and the conjugate operation on a given signal. Combining both channels is done by:

(3) $$\text{EndCorr}=\sqrt{{\text{Corr}}_{\frac{\pi }{2}\text{rad}}^{2}+{\text{Corr}}_{0\text{rad}}^{2}}$$

where *EndCorr*, ${\text{Corr}}_{\frac{\pi }{2}\text{rad}}$ and *Corr*_0_*_rad_* respectively denote the final correlation result, the correlation shifted branch and the in phase branch. Figure 6 illustrates the expected shape of the correlation result. The time difference between A and B corresponds to the time necessary for sound to travel from the sender to the receiver. Point A corresponds to the synchronization time between sender and receiver, while B corresponds to the time at the maximum correlation value. Figure 7 gives an overview of the DSSS correlator.

#### 3.3.2. FHSS Ranging

For ranging with FHSS, we developed a new approach based on finite impulse response (FIR) [19] filtering. The input signal contains four different frequencies, which can be separated from each other by applying a bandpass filter around each of the desired frequencies (*i.e.*, 36.684 kHz, 40.960 kHz, 46.080 kHz and 51.200 kHz). The lengths of both the high-pass and low-pass filters are set to 32. These lengths are a tradeoff between the filter steepness and the calculation overhead. A combination of a low-pass and a high-pass FIR filter is applied at each frequency band. Four such filter systems are used in parallel in order to be able to filter around the four frequencies simultaneously.

**Figure 8.**
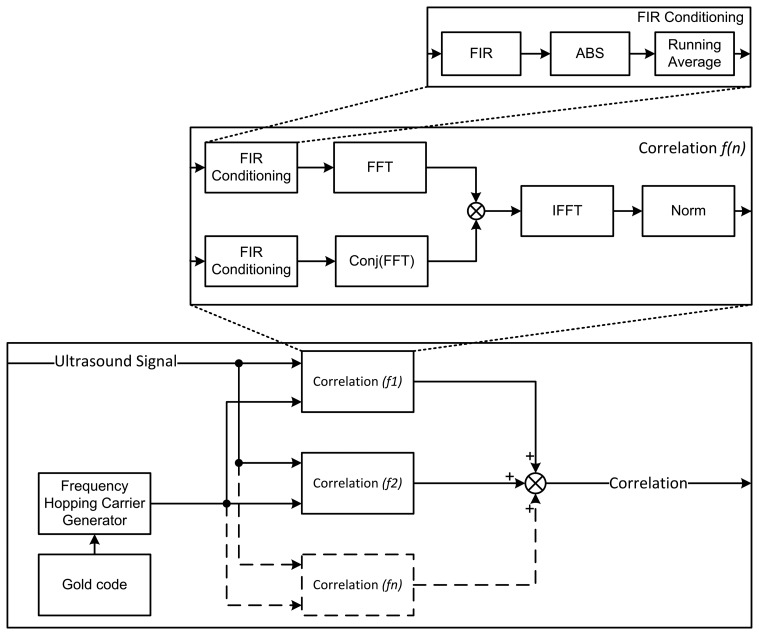
Frequency hopping signal correlator. Where FIR, ABS, IFFT and Conj respectivily denote the finite impulse response filter, the absolute value, the inverse fast Fourier transform and the conjugate operations on a given signal.

After filtering, the absolute value (ABS) is taken from the signal, followed by a running average. The latter smooths the signals, from which a correlation with the reference signal can be taken. The correlation method is similar to the one used in DSSS. The complete time correlation process is shown in Figure 8.

## 4. Ranging Analysis

**Figure 9.**
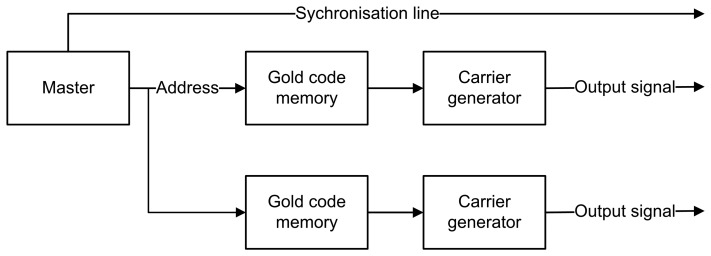
FHSS sender logic with multiple outputs and one synchronization line.

**Figure 10.**
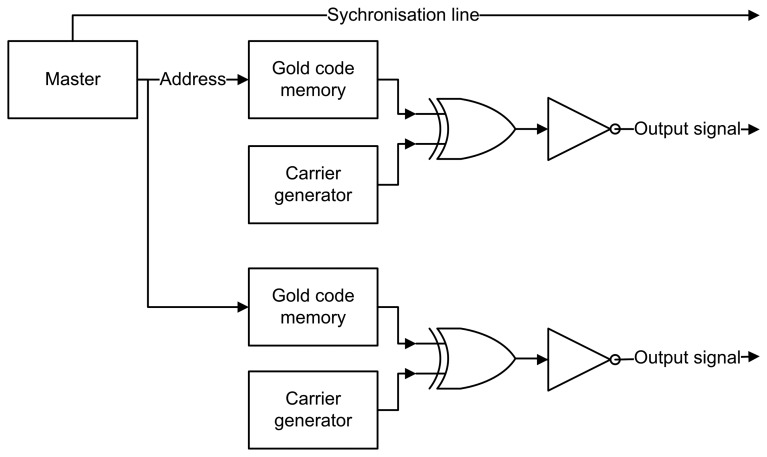
DSSS sender logic with multiple outputs and one synchronization line.

The ranging calculations for both DSSS and FHSS are first applied by using one sender and one receiver. This test allows us to determine the accuracy and precision of the system without additional noise. In the second step, two senders are simultaneously sending ultrasound coded signals, which are than processed by the receiver. The influence of additional noise induced by an additional sender is studied. During these tests, we limited ourselves to only two senders. A major problem for the duplication of the sender's hardware is the electrostatic transducer. This transducer needs a high operating voltage while offering a small beamwidth. The advantage, however, is the wideband capabilities. We implemented a dual channel DSSS and FHSS sender logic onto the FPGA. However, to reduce the amount of required synchronization wires between the senders and receiver (using the PicoScope 2203), the complete system is synchronized using only one master module. The senders’ logic for FHSS and DSSS is given in Figures 9 and 10.

Some aspects need to be taken into account during the ranging process. The first aspect concerns the sample *versus* distance ratio. As the distance between sender and receiver increases, the number of samples starting from the synchronization signal increases. Linear regression is therefore used to determine the sample to distance ratio. Another aspect consists in the theoretical maximal available precision of the system. In a previous section, we mentioned that the PC oscilloscope digitizes the input signal at a rate of 250 kSps. The precision of the system is limited by this sampling rate and cannot be shorter than the duration of one sample. The highest available precision available is therefore given by:

(4) $$p=\upsilon_{\text{sound}}\cdot t$$

where *p* is the precision margin in meters, *υ_sound_* the velocity of sound and *t* the time between two successive samples. At 20 °C, this margin is equivalent to about 1.4 mm. Signal loss and noise can cause the receiver to have unreliable ranging outputs. In order to be able to detect faulty measurements in our experiments, each measurement is repeated 250 times. Furthermore, the normal distribution model (*i.e.*, 95% error rate) is applied together with the absolute error against the real distance (*i.e.*, accuracy). The measurements are taken with an interval of 20 cm, starting from 0 cm going up to 500 cm.

### 4.1. Single-Access Ranging

**Figure 11.**
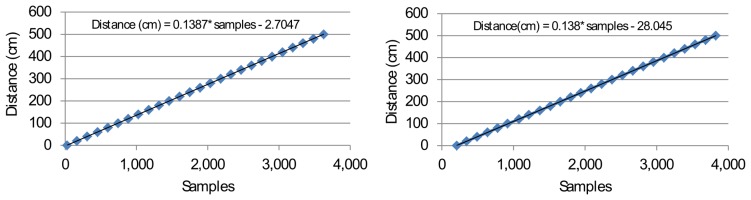
DSSS (**left**) and FHSS (**right**) sample to distance plot, with the linear best fit regression.

The first set of measurements concerns distance ranging in which only one sender is used with one receiver (*i.e.*, single-access). Both DSSS and FHSS experiments show a linear relation between the number of samples and the distance (Figure 11).

**Figure 12.**
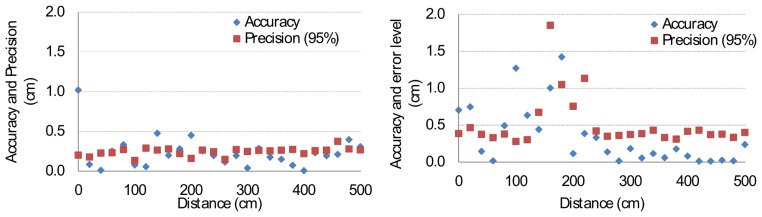
Accuracy and precision (95 % interval) of DSSS (**left**) and FHSS (**right**).

The accuracy (*i.e.*, absolute error rate) and precision (95% confidence interval) remain generally below one centimeter (Figure 12). The FHSS method, however, presents less precise readings than the DSSS method. This is probably caused by the running average during the signal processing, which smooths (spreads) the signal over time.

In both cases, no abnormalities were measured and no outliers were eliminated. Although the sample to distance coefficient is almost equal in both methods, the offset differs for both methods. The FHSS method uses FIR filtering and the running average, which cause a sample delay during the processing. This delay is reflected in the offset.

### 4.2. Multiple-Access Ranging

In the second set of the measurements, two senders and one receiver are used (*i.e.*, multiple-access). Both senders are simultaneously sending an ultrasound coded signal (with unique orthogonal code), while the receiver calculates the distance against both senders. Measurements are taken with one sender as a mobile node, while the other sender is kept at a distance of 200 cm from the receiver. The distance of the mobile node against the receiver ranges linearly from 0 cm to 500 cm in steps of 20 cm. The receiver remains at a fixed position.

**Figure 13.**
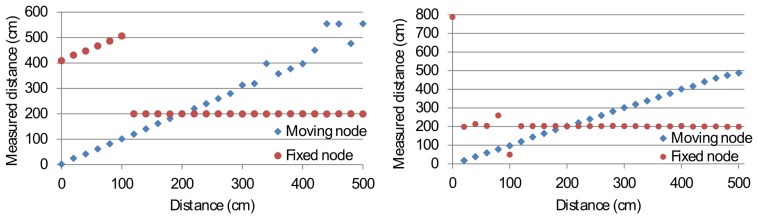
DSSS (**left**) and FHSS (**right**) multiple-access ranging.

**Figure 14.**
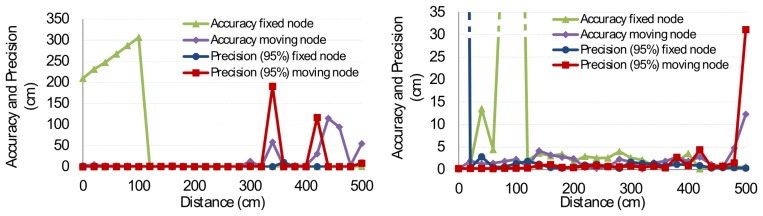
Accuracy and error levels of DSSS (**left**) and FHSS (**right**) in the multiple access setup. Due to scaling problems, we limited the graph of FHSS to an accuracy and precision level of 35 cm. Results that are outside of the graph are marked with a dashed line.

In the case of two senders, one can define three zones of measurements. The zones are defined according to the distance between the mobile node and the receiver. The first zone concerns the distance range in which distances to both senders are successfully calculated. This zone ranges from 120 cm to 300 cm. The other zones range from 0 cm to 120cm and beyond 300 cm. The major difference between the first zone and the other zones is given in the distance ratio. In the former case, the ratio of both senders against the receiver is close to 100%, meaning that both signals arrive with similar strength at the receiver. The signal-to-noise ratio of both signals is thus sufficient to be measured well by the receiver. In the other case, the signal-to-noise ratio decreases and is even dominated by the signal of the closest node. The resulting distance measurements are shown in Figure 13. One can notice that the FHSS method has, on average, a more robust distance measurement than DSSS. This is especially the case in the third zone, where the FHSS method remains stable at a distance up to 460 cm. However, the overall accuracy and precision of the DSSS method are generally better than FHSS (Figure 14).

The accuracy and precision loss occurs when one sender is far from the receiver compared to the closest sender. The domination of one signal by the other can clearly be seen in the case of DSSS, where the farthest sender remains at a constant distance from the closest sender. In this case, the signal of the more distant sender is cross-correlating with the signal of the closest sender. This phenomenon is also known as the near-far problem. FHSS is more robust in a multiple-access environment. This is due to the fact that FHSS is spread over multiple frequencies, and thus, collisions on a given frequency band are less likely to happen.

Some outliers were detected during this set of measurements. These outliers were easily detected and can be categorized into two classes. The first class concerns the outliers with a negative distance. The second class concerns distances that are out of range (*i.e.*, 30m or more). The amount of outliers remains stable during the measurements and concerns less than 25 of the 250 measurements.

A possible solution would be to use the noise cancellation approach, where the strongest signal is first correlated and subtracted from the original signal. The remaining signal can be correlated against the more distant sender's signal. Another method to remove unreliable measurements can be done by using multilateration against multiple senders. The receiver is able to apply multilateration when four or more senders can be detected. When a distance estimate against a sender does not match with the other ranges, it can simply be eliminated. A combination of both DSSS and FHSS could also be used in order to combine the benefits of both methods, where DSSS is modulated onto FHSS. The expected results of this hypothesis are, however, uncertain.

## 5. Conclusion and Future Work

In this paper, we investigated a new wideband ultrasound ranging method using frequency hopping spread spectrum and finite impulse response filters. We compared this method against the existing direct sequence spread spectrum technique. We used the electrostatic instrumental grade 600 transducer [17] for the sender, while the MEMS SPM0404UD5 [16] transducer was chosen for the receiver. Additional hardware interfaces were designed for both the sender and receiver. The appropriate sender and receiver logic were also developed in order to apply the ranging techniques.

Results show that in a single-access environment, the DSSS performs slightly better than the FHSS approach in terms of accuracy and precision. However, the obtained accuracy and precision levels for FHSS generally remain below 1 cm. When DSSS and FHSS are applied in a multiple-access environment, a better overall reliability is obtained with the FHSS method. The latter is due to the multiple frequency bands used in the method.

In order to retrieve the upper-bound of the achievable localization accuracy, we have used a physical wire to synchronize the nodes in the test setup. In a real-world setup, the synchronization will be done by means of a wireless link. We specified the maximum synchronization error that might be induced by a wireless link. In our case, the accuracy remains below one centimeter, which means that a maximum synchronization-jitter of 1.5 ms is allowed.

The next steps towards a fully operational system will include the use of more senders and the use of a transducer with a larger beam angle than the currently used one by the senders. Previously mentioned techniques, like multilateration, noise cancellation and other modulation schemes, will also be investigated towards a reliable indoor localization system. The feasibility of porting the ranging calculations of the receiver onto FPGAs will also be investigated.

## Figures and Tables

**Table 1.** Modulation truth table.

**Carrier (C)**	**Gold code (G)**	**Output (O)**
0	0	1
0	1	0
1	0	0
1	1	1

**Table 2.** Summary of the XC3S400 area utilization. FHSS—frequency hopping spread spectrum; LUT—lookup table; IO—input-output.

**XC3S400-FT256**	**Total Available**	**Used DSSS**	**Used FHSS**
Slices	3584	48 (1.4%)	57 (1.6%)
LUTs	7168	69 (1%)	97 (1.4%)
IO buffers	173	3 (1.7%)	3 (1.7%)
